# Editorial on the Special Issue: “Advances in Retinal Image Processing”

**DOI:** 10.3390/jimaging11100366

**Published:** 2025-10-16

**Authors:** P. Jidesh, Vasudevan Lakshminarayanan

**Affiliations:** 1 Department of Mathematical and Computational Sciences, National Institute of Technology Karnataka, Surathkal 575025, Karnataka, India; 2 Theoretical and Experimental Epistemology Laboratory, University of Waterloo, Waterloo, ON N2J 4A8, Canada

Retinal disorders are one of the major causes of visual impairment. The damage to the retina can lead to severe visual challenges that can eventually cause blindness [1]. The lack of available early detection facilities is one of the challenges in handling this problem. Prompt diagnosis of a retinal disorder can reduce the risk of visual impairment in many cases [2]. Two of the prominent retinal imaging modalities that are widely used for retinal examination are fundus images and optical coherence tomographs. Fluorescent angiography is yet another non-invasive imaging modality used for retinal analysis [3]. An ophthalmologist analyzes these images to arrive at a diagnosis. Due to the large patient populations, especially in developing countries, a proper diagnosis becomes a challenging task for the clinicians. Moreover, a lack of adequate facilities for acquiring images, especially in remote areas, is another challenge for the timely diagnosis of disorders. Automated retinal analysis can handle large amounts of data with minimal manual intervention. The advent of AI and machine/deep learning modalities in the last decade has changed the face of the scientific world. Retinal image analysis has also witnessed commendable progress due to the advancement of AI and ML in the present context [4], with AI and ML models becoming more efficient and accurate [5]. Convolutional Neural Networks (CNNs) and their variants are also employed in retinal imaging applications [6]. A further development is reported in [7], wherein the authors go one step further and utilize transformer networks for retinal image processing. The ability of these models to handle large amounts of input data in a short time has revolutionized the domain of retinal image processing. The domain of retinal image enhancement and registration, multi-modal analysis, and multiple disorder detection are the current focuses of retinal imaging, and AI models are employed extensively in their implementations.

In this regard, this Special Issue focuses on multiple topics related to the retinal image acquisition, analysis, and processing (https://www.mdpi.com/journal/jimaging/special_issues/6OWLNIZNK8, accessed on 10 October 2025). Nine contributory papers are included as a part of this Special Issue. Among them, eight are research articles and one is a comprehensive review article. Each of these papers discuss various cutting-edge technologies proposed for various retinal imaging and image processing applications. All of these papers have undergone several rounds of rigorous review before being considered for publication. A brief description of the papers accepted for the Special Issues is highlighted below.

The first contributory paper, titled “Robust PCA with *L*_*w*,∗_ and *L*_2,1_ Norms: A Novel Method for Low-Quality Retinal Image Enhancement”, is related to retinal image pre-processing and enhancement for efficient analysis and detection of disorders. The authors have studied the role of Principal Component Analysis (PCA) with different norm constraints for enhancing low-quality retinal images that are degraded in their row forms in many applications. As claimed by the authors, their method introduces a novel parameter update approach and significantly improves retinal image quality, detecting cataracts and diabetic retinopathy.

A retinal image quality assessment technique is studied in the second contributory article titled “Subjective Straylight Index: A Visual Test for Retinal Contrast Assessment as a Function of Veiling Glare”. A contrast assessment of retinal images was performed as a function of Veiling Glare. Contrast assessments are vital for analyzing and making a diagnosis from the images. If the image quality hampers the diagnosis and detection process it can eventually result in spurious analysis. The authors propose a new computational methodology to generate visual acuity charts affected by ocular scattering effects. A retinal contrast assessment is analytically studied in this work.

In the third contributed paper titled “When Sex Matters: Differences in the Central Nervous System as Imaged by OCT through the Retina”, the authors compute mean value fundus images for the neuroretina layers as imaged via OCT of healthy individuals. Texture metrics were obtained from these images to assess whether women and men have the same retina texture characteristics in both eyes. The authors arrive at the conclusion that the differences between the right and left eyes manifest differently in females and males.

The main objective of the fourth contribution, titled “Arteriovenous Length Ratio: A Novel Method for Evaluating Retinal Vasculature Morphology and Its Diagnostic Potential in Eye-Related Diseases”, is to propose a new method for assessing one of the morphological changes in the fundus through morphometric analysis of retinal images. The proposed method in this paper introduces a novel approach called the arteriovenous length ratio (AVLR). Unlike commonly used measures such as the arteriovenous width ratio or tortuosity, the AVLR focuses on assessing the relative length of arteries and veins in the retinal vasculature.

The fifth contributory paper, titled “Retinal Microvasculature Image Analysis Using Optical Coherence Tomography Angiography in Patients with Post-COVID-19 Syndrome”, highlights the analysis of retinal microvasculature changes in patients after COVID-19. In this study, the authors investigated the retinal microvasculature in PCS patients using OCT-angiography and analyzed the macular retinal nerve fiber layer (RNFL) and ganglion cell layer (GCL) thickness via spectral domain-OCT (SD-OCT).

In the sixth contributory paper titled “Evaluating Retinal Disease Diagnosis with an Interpretable Lightweight CNN Model Resistant to Adversarial Attacks”, the authors propose a lightweight CNN model for retinal disease diagnosis evaluation. Given the computational requirement of a CNN model, a lightweight model is required to handle large volumes of data efficiently. The advent of deep learning frameworks has changed the face of retinal image analysis. This OCT-based model built on a CNN framework has been found to work efficiently in diagnosing retinal diseases.

An early screening and diagnosis of retinal diseases can be performed from optical coherence tomography (OCT) images using deep learning methods. In the seventh contributory paper titled “Multi-Fundus Diseases Classification Using Retinal Optical Coherence Tomography Images with Swin Transformer V2”, the authors propose a shifting window-based transformer for multi-fundus disease classification using OCT images. The transformers efficiently attend to important features as they use a multi-headed attention network for feature enhancement. Furthermore, the Swin transformers improve their computational efficiency.

In the eighth contributory paper titled “Advancements in Cataract Detection The Systematic Development of LeNet-Convolutional Neural Network Models”, the authors portray various advancements in cataract detection using a CNN-based model. Despite the fact that cataracts can be diagnosed and treated successfully, patients often delay seeking medical attention due to the relatively asymptomatic nature of the disease. To address this challenge, this research focuses on the identification of cataract abnormalities using image processing techniques and machine learning for preliminary assessment.

Finally, in the ninth and final paper titled “Denoising of Optical Coherence Tomography Images in Ophthalmology Using Deep Learning: A Systematic Review”, a systematic survey of denoising OCT images under a deep learning framework is carried out. This is a review contribution where the authors have completed an elaborative analysis on various state-of-the-art denoising methods for OCT image restoration. The efficiency of deep learning frameworks in handling the denoising task make them a better choice compared to traditional restoration models.

## References

[1] Abràmoff M.D., Garvin M.K., Sonka M. (2010). Retinal imaging and image analysis. IEEE Rev. Biomed. Eng..

[2] Early Treatment Diabetic Retinopathy Study Research Group (1991). Early photocoagulation for diabetic retinopathy. ETDRS Rep. 9.. Ophthalmology.

[3] Baudoin C.E., Lay B.J., Klein J.C. (1984). Automatic detection of microaneurysms in diabetic fluorescein angiography. Rev. Epidemiol. Sante Publique.

[4] Bahr T., Vu T.A., Tuttle J.J., Iezzi R. (2024). Deep Learning and Machine Learning Algorithms for Retinal Image Analysis in Neurodegenerative Disease: Systematic Review of Datasets and Models. Transl. Vis. Sci. Technol..

[5] Patil A.D., Biousse V., Newman N.J. (2022). Artificial intelligence in ophthalmology: An insight into neurodegenerative disease. Curr. Opin. Ophthalmol..

[6] Bhandari M., Shahi T.B., Neupane A. (2023). Evaluating Retinal Disease Diagnosis with an Interpretable Lightweight CNN Model Resistant to Adversarial Attacks. J. Imaging.

[7] Li Z., Han Y., Yang X. (2023). Multi-Fundus Diseases Classification Using Retinal Optical Coherence Tomography Images with Swin Transformer V2. J. Imaging.

